# Fostering Intergenerational Connections in the time of Covid-19: A Friendly Caller Program

**DOI:** 10.1093/geroni/igab046.3136

**Published:** 2021-12-17

**Authors:** Maria Claver, Alexandra Wilkinson, Tatia Clark, Chan Park

**Affiliations:** 1 California State University, Long Beach, Whittier, California, United States; 2 California State University, Long Beach, California State Univ Long Beach, California, United States; 3 California State University Long Beach, California State University, Long Beach, California, United States; 4 Covia - Lutheran Towers, Long Beach, California, United States

## Abstract

Social isolation affects one in five older adults, significantly increases the risk of premature death from all causes and is associated with higher rates of depression, anxiety and suicide. Covid-19 has exacerbated social isolation, including among older adults that reside in senior apartments. In response, a Friendly Caller Program was developed to foster intergenerational social connections among university students and residents in a large housing community that serves older adults aged 62 and older who have limited income and have mobility impairments. This study aimed to evaluate the Friendly Caller Program from the perspective of the older adult. An online survey includes questions about the participants’ demographic characteristics, physical and mental health self-assessment, social support, and ways in which the Friendly Caller Program has affected these areas of their life. An open-ended question assesses older adult participant expectations of the Friendly Caller Program. Results describe the population currently being served by this program and indicate that the program has a positive influence on participants’ feelings of safety, support and ability to function. Suggestions for future research include assessing university student perceptions about the benefits of participation as callers in the program and creating a toolkit that can guide other universities to create similar programs in partnership with housing communities that serve older adults.

